# Late-day intraocular pressure–lowering efficacy and tolerability of travoprost 0.004% versus bimatoprost 0.01% in patients with open-angle glaucoma or ocular hypertension: a randomized trial

**DOI:** 10.1186/1471-2415-14-151

**Published:** 2014-11-28

**Authors:** Harvey B DuBiner, Douglas A Hubatsch

**Affiliations:** Clayton Eye Center, 1000 Corporate Center Drive, Suite 102, Morrow, GA 30260 USA; Alcon Laboratories, Inc., Fort Worth, TX USA

**Keywords:** Travoprost, Bimatoprost, Prostaglandin analog, Intraocular pressure, Hyperemia

## Abstract

**Background:**

Medications to control intraocular pressure (IOP) are frequently preserved using benzalkonium chloride (BAK), which can negatively affect the ocular surface. Data are needed to assess efficacy and safety of prostaglandin drugs preserved with and without BAK. The present study compared the efficacy and safety of BAK-free travoprost 0.004% (TRAV) and BAK 0.02%–preserved bimatoprost 0.01% (BIM) during late-day time points in patients with open-angle glaucoma or ocular hypertension.

**Methods:**

This was a 12-week, phase 4, randomized, investigator-masked, crossover study. 84 patients with IOP ≥24 and <36 mmHg were randomized 1:1 to receive once-daily TRAV or BIM for 6 weeks followed by an additional 6-week crossover period. IOP was measured at the end of each treatment period at 4, 6, and 8 pm. TRAV was considered noninferior to BIM if the upper limit of the 95% CI of the between-group difference in mean IOP was ≤1.5 mmHg. Adverse events were assessed throughout the study.

**Results:**

One patient discontinued due to allergic conjunctivitis, and 2 patients with missing data were excluded; 81 patients were included in the per-protocol population (mean ± SD age, 58.3 ± 11.4 years; TRAV/BIM, n = 41; BIM/TRAV, n = 40). After 6 weeks, mean IOP with TRAV (17.4 ± 2.7 mmHg; change from baseline, -6.0 mmHg) was similar to BIM (17.2 ± 2.6 mmHg; change from baseline, -6.3 mmHg); the between-group difference was 0.22 mmHg (95% CI, -0.22 to 0.67). Thus, noninferiority of TRAV versus BIM was demonstrated. Mean IOP at each time point and mean and percentage IOP change from baseline were not significantly different between treatments. All treatment-emergent adverse events were mild to moderate. The incidences of mild ocular hyperemia with TRAV and BIM were 31% and 39%, respectively; moderate hyperemia was observed in 2% of patients receiving BIM.

**Conclusion:**

Late-day IOP-lowering efficacy of BAK-free TRAV was noninferior to that of BAK 0.02%–preserved BIM; both reduced baseline IOP by 25%. Both treatments were well tolerated, although a higher incidence of moderate ocular hyperemia was observed with BIM.

**Trial registration:**

ClinicalTrials.gov identifier, NCT01464424; registered November 1, 2011.

## Background

Glaucoma is the second-leading cause of blindness worldwide and is a chronic, progressive disorder [1]. Elevated intraocular pressure (IOP) has been associated with increased risk [2] and progression [3, 4] of glaucoma, including loss of visual field [2–4] and optic disc deterioration [2]. Because of this, lowering IOP through mechanical (surgical/laser) or pharmaceutical means is the primary therapeutic recommendation of the American Academy of Ophthalmologists [1]. Lowering IOP at a single time point may not adequately reflect changes in peak IOP because of daily fluctuations in IOP. Indeed, the time of highest IOP may vary among individuals, with the peak occurring at a time in the 24-hour period other than that typically measured during clinical trials [5]. Thus, consistent 24-hour control of IOP is essential.

In decades past, β-blockers were the most commonly prescribed IOP-lowering medication for patients with glaucoma; however, use of prostaglandin analogs (e.g., travoprost, bimatoprost, and latanoprost) has increased in recent years such that they are now the dominant IOP-lowering medication [6]. In contrast to β-blockers that decrease IOP by reducing aqueous flow [7], prostaglandin analogs exert their effects by increasing uveoscleral [7–9], and perhaps trabecular [9], aqueous flow. This mechanistic difference results in a greater IOP-lowering effect than that observed with β-blockers [10], an improved systemic side-effect profile, and a concomitant increase in patient adherence [11]. However, conjunctival hyperemia [11, 12], eyelash changes [12], induced iris darkening [12, 13], and periocular skin pigmentation [12, 13] have been associated with prostaglandin analogs.

Bimatoprost is a prostamide (i.e., a synthetic analog of fatty acid amides) indicated for reducing IOP in patients with open-angle glaucoma or ocular hypertension [14]. In a 12-month pooled analysis of 2 randomized head-to-head clinical trials, bimatoprost 0.03% preserved with 0.005% benzalkonium chloride (BAK) reduced IOP to a greater extent than the β-blocker timolol, with mean percentage IOP reductions from baseline ranging between 30% and 33% with bimatoprost compared with 20% and 23% with timolol [15]. Subsequent 2-year [16] and 4-year [17] extension studies demonstrated sustained IOP-lowering efficacy with bimatoprost 0.03% versus timolol, with a higher incidence of ocular hyperemia reported with bimatoprost 0.03% (6% - 14%) versus timolol (0% - 3%). To improve tolerability, the concentration of bimatoprost was lowered and the concentration of BAK increased 4-fold to produce a new BAK 0.02%–preserved bimatoprost 0.01% (BIM) formulation [18]. This new formulation demonstrated IOP-lowering efficacy slightly lower than that of bimatoprost 0.03% after 12 months of treatment (range of BIM IOP change from baseline, -5.2 to -7.8 mmHg; bimatoprost 0.03% range, -5.6 to -8.0 mmHg) [18] and further increased patient adherence [19]. The incidence of conjunctival hyperemia, the most common adverse event (AE) reported with prostaglandin analogs [20], was nonsignificantly lower with BIM (29% of patients) compared with bimatoprost 0.03% (37% of patients) [18].

Travoprost 0.004% is a selective agonist for the prostaglandin F receptor that reduces IOP in patients with glaucoma or ocular hypertension to a significantly greater extent than timolol [20]. Travoprost provides increased IOP-lowering efficacy for patients who were previously uncontrolled on other IOP-lowering therapies [21, 22]. In 2001, BAK-free, sofZia® (Alcon Laboratories, Inc., Fort Worth, TX)–preserved travoprost 0.004% (TRAV) became available as an alternative therapy for patients with concomitant ocular surface disease and those with sensitivity to BAK [23]. In a head-to-head 3-month comparative study, the IOP-lowering effect of TRAV administered once daily in the evening was noninferior to travoprost 0.004% throughout a 24-hour period and was associated with fewer incidences of ocular hyperemia (TRAV, 6%; travoprost 0.004%, 9%) [23]. Use of TRAV also resulted in fewer ocular AEs than BAK-preserved bimatoprost or latanoprost [24, 25] and was preferred over bimatoprost [25] and latanoprost [25, 26] by patients. Overall, BAK-free TRAV was associated with similar IOP lowering and fewer adverse ocular surface effects than other BAK-preserved prostaglandins [24, 25]. Furthermore, an integrated analysis demonstrated that both BAK-preserved travoprost 0.004% and BAK-free TRAV provide IOP control for up to a full day [27].

The objective of the present study was to compare the IOP-lowering efficacy and tolerability of BAK-free TRAV versus BAK 0.02%–preserved BIM during late-day time points (4, 6, and 8 pm) and to demonstrate noninferiority of TRAV versus BIM efficacy in patients with open-angle glaucoma or ocular hypertension after 6 weeks of treatment.

## Methods

### Patients

Eligible patients were ≥18 years of age with a clinical diagnosis of open-angle glaucoma or ocular hypertension in ≥1 eye, baseline IOP ≥24 and <36 mmHg in the study eye at 8 am ±30 minutes after washout of IOP-lowering medication at 2 eligibility visits, and a best corrected visual acuity (BCVA) of 20/100 or better in each eye. Patients must have been willing to discontinue other ocular hypotensive medications during the study and have, in the opinion of the investigator, IOPs within a safe range.

Key exclusion criteria included a history of allergy, hypersensitivity, or low tolerance to components of TRAV or BIM; abnormalities preventing reliable applanation tonometry and examination of the anterior chamber; intraocular conventional or laser surgery <3 months before screening; progressive retinal or optic nerve disease; or use of systemic medications known to affect IOP, unless on a stable regimen for ≥7 days before screening. Patients who could not safely discontinue use of all IOP-lowering medications for a minimum period of 3 ± 1 to 28 ± 1 days before enrollment, had participated in any other investigational study ≤30 days before screening, or had used any systemic (oral), injectable, or topical steroids were also excluded.

### Study design and treatment

This phase 4, randomized, prospective, crossover, investigator-masked, controlled study was conducted by a single investigator at 2 US investigational centers (ClinicalTrials.gov identifier, NCT01464424) from October 2011 to June 2012. Investigators and study personnel were masked to patient information and treatment groups. The study received institutional review board approval from Institutional Review Board Services (Independent Central Institutional Review Board, Aurora, Ontario, Canada) and complied with the ethical standards set forth by the Declaration of Helsinki and Good Clinical Practice. All patients provided written informed consent before any screening procedures were performed.

The study consisted of 2 consecutive 6-week study periods without an intervening washout phase. In the first phase, patients were randomized 1:1 to TRAV (TRAVATAN Z®, Alcon Laboratories, Inc.) or BIM (Lumigan®, Allergan, Irvine, CA); in the second phase, patients were crossed over to the alternate treatment. Randomization was performed by independent biostatisticians (Howard M. Proskin & Associates; Rochester, NY) using a random variate generator (SAS, release 9.1; SAS Institute, Cary, NC) that assigned patients to 1 of 2 treatment groups (TRAV to BIM, BIM to TRAV) in 25 successive blocks, with 2 patients from each treatment group in each block. Phase 1 consisted of 4 study visits: 1 screening visit, 2 eligibility visits, and 1 follow-up visit after 6 weeks of treatment with the first medication. At the screening visit, patients were evaluated for eligibility and asked to discontinue use of all IOP-lowering medications before returning for the first eligibility assessment. The 2 eligibility visits occurred 3 to 8 days apart. During these visits, adherence to the medication washout period and any changes in concomitant medications were documented. Patients who qualified for the study (i.e., had IOP ≥24 and <36 mmHg at 8 am) had 1 eye designated as the study eye during the second eligibility visit. If only 1 eye met the IOP criteria and was to receive study medication, that eye was appointed the study eye; if both eyes were to receive medication, the eye with the highest IOP at baseline (i.e., the second eligibility visit) was the study eye; and if baseline IOP was equal in both eyes, the right eye was designated as the study eye. Eligible patients received 2 bottles of appropriate study medication and were instructed to administer the medication to the study eye once daily at 8 pm for 6 weeks.

The first study visit occurred in the afternoon approximately 6 weeks ±3 days after the second eligibility visit; the second study visit occurred approximately 6 weeks ±3 days after the first study visit (i.e., study week 12). The date and time of the last instillation of the study medication were documented at both study visits; if a patient had not administered the medication during the evening before the study visit, the visit was rescheduled. During the first study visit, the second crossover medication was dispensed with directions for administration identical to that of the first study medication, once daily at 8 pm for 6 weeks.

Changes in medical health and concomitant medications, BCVA, slit lamp, IOP measurements, and ocular hyperemia grading were assessed at all visits. BCVA was performed using a Snellen visual acuity chart. Slit-lamp biomicroscopy was used to evaluate ocular signs (e.g., corneal, lens, eyelids/conjunctiva, iris/anterior chamber) before all IOP measurements. IOP was assessed at 8 am ±30 minutes at the screening and first and second eligibility visits, and at 4, 6, and 8 pm ±30 minutes at the second eligibility visit (baseline) if the IOPs qualified. During all study visits, Goldmann applanation tonometry was used. Solicited and unsolicited AEs were recorded at all study visits.

### Outcomes

The primary efficacy endpoint was comparison of mean IOP averaged across 3 late-day time points (4, 6, and 8 pm) after 6 weeks of treatment to demonstrate noninferiority. Secondary endpoints were between-group comparison of mean IOP values at each 4, 6, and 8 pm time point; differences in mean change in IOP from baseline; and percentage change from baseline. Exploratory analyses evaluated between-group differences in conjunctival hyperemia severity after 6 weeks of treatment. Solicited and unsolicited AEs and serious AEs were collected throughout the study and were coded using the Medical Dictionary for Regulatory Activities Version 15.0. AEs were associated with the treatment administered most recently before the time the event was reported. Treatment-emergent AEs were those that occurred on or after the date of first drug administration.

### Data analysis and statistics

Efficacy analyses were performed in the study eye in the intent-to-treat (ITT) population (i.e., all patients receiving study medication and having ≥1 on-therapy study visit) and the per-protocol (PP) population (i.e., all patients receiving study medication, completing all study visits per protocol timelines and criteria, and satisfying inclusion/exclusion criteria). Safety data, including conjunctival hyperemia severity, were analyzed in the safety population (i.e., all patients receiving study medication). Efficacy data for the PP population are presented.

Efficacy parameters were analyzed using an analysis of variance (ANOVA) model that included sequence, period, and treatment as fixed factors and patient within sequence as a random factor. A 2-sided 95% CI was constructed for the difference between mean IOP values for the 2 study treatments. Noninferiority of TRAV compared with BIM was supported if the upper limit of the 95% CI for the between-treatment difference in mean IOP was ≤1.5 mmHg. For exploratory statistical analyses, ocular hyperemia scale ordinal response data were converted to a numerical scale: 0 = none/trace, 1 = mild, 2 = moderate, 3 = severe. Differences in ocular hyperemia from baseline to study completion were evaluated using Wilcoxon signed-rank tests. Between-group occurrences of rates of AEs were compared using McNemar tests. All statistical analyses were performed by an independent biostatistician using SAS, release 9.1.3 or higher (SAS Institute).

Based on an assumed between-patient SD of 3.0 mmHg across treatment groups, a within-patient correlation of 0.5 mmHg between IOP values, and a mean IOP for TRAV less than or equal to BIM, a sample size of 74 patients was calculated to provide 80% power to detect a mean IOP difference of 1.5 mmHg between groups.

## Results

### Patients

**Figure 1:**
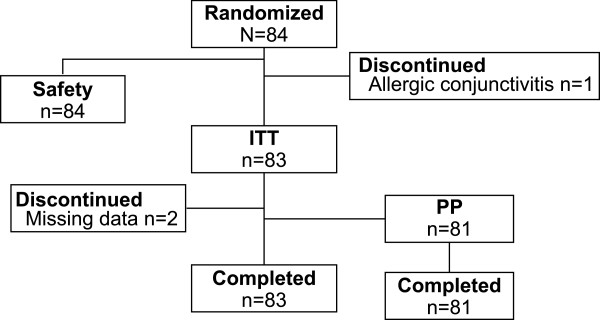
**Patient flow.** ITT = intent to treat; PP = per protocol.

A total of 84 patients were enrolled, randomized to treatment, and included in the safety population (TRAV followed by BIM [TRAV/BIM], n = 42; BIM followed by TRAV [BIM/TRAV], n = 42; Figure 1). Of these, 1 patient in the BIM/TRAV group discontinued during the first study period (BIM) because of an AE (allergic conjunctivitis); therefore, 83 patients were included in the ITT dataset (TRAV/BIM, n = 42; BIM/TRAV, n = 41). Two patients had missing data at the IOP measurement visits and were excluded from the PP population; thus, the PP population consisted of 81 patients (TRAV/BIM, n = 41; BIM/TRAV, n = 40). The mean age ± SD of the PP population was 58.3 ± 11.4 years (range, 26–81). Most patients were women (67%; n = 54/81) and black (62%; n = 50/81; Table 1).

**Table 1:** **Patient demographics**

	Per-protocol population
Demographic	(n = 81)
Age	
Mean ± SD, y	58.3 ± 11.4
Race, n (%)	
Black	50 (62)
White	28 (34)
Asian	3 (4)
Sex, n (%)	
Male	27 (33)
Female	54 (67)

### Efficacy

**Figure 2:**
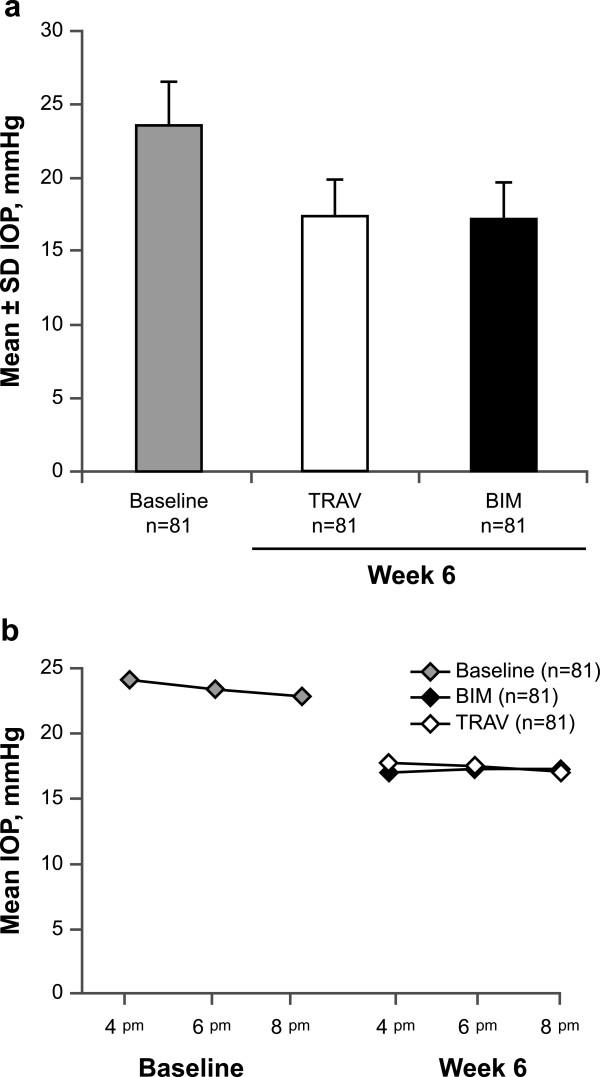
**Mean IOP at baseline and after 6 weeks of treatment with TRAV vs BIM. (a)** Mean ± SD IOP across time points (i.e., mean of 4, 6, and 8 pm assessments). **(b)** Mean IOP at individual time points (TRAV, n = 80 at the 4 and 8 pm time points). BIM = bimatoprost 0.01%; IOP = intraocular pressure; TRAV = benzalkonium chloride–free travoprost 0.004%.

In the PP population, mean late-day IOP at baseline was 23.5 ± 3.2 mmHg. After 6 weeks of treatment, mean late-day IOP was similar between TRAV (17.4 ± 2.7 mmHg) and BIM (17.2 ± 2.6 mmHg; Figure 2A). The between-group difference in mean IOP was 0.22 mmHg (95% CI, -0.22 to 0.67). Because the upper limit of this 95% CI was ≤1.5 mmHg, the IOP-lowering efficacy of TRAV was found to be noninferior to that of BIM.

Mean IOP measurements at each late-day time point (4, 6, and 8 pm) after 6 weeks of treatment were similar and ranged between 17 and 18 mmHg. Mean IOP was similar between TRAV and BIM at each time point (Figure 2B). The largest between-group difference occurred at the 4 pm time point (between-group difference, 0.61 mmHg [95% CI, -0.03 to 1.26]), but this difference was not significant (*P* = 0.062). Changes in IOP from baseline after 6 weeks of treatment were also not significantly different between TRAV and BIM at all time points. IOP reduction with TRAV ranged from -6.4 mmHg at 4 pm to -5.8 mmHg at 8 pm (overall mean IOP ± SD reduction, -6.0 ± 3.2 mmHg). Similar reductions were observed with BIM (-7.0 mmHg at 4 pm to -5.7 mmHg at 8 pm; overall mean IOP reduction, -6.3 ± 2.8 mmHg). Similarly, the overall percentage IOP reduction from baseline was not significantly different between TRAV and BIM (-25% and -26%, respectively; between-group comparison, *P* = 0.25). Percentage change from baseline at each time point was 26%, 24%, and 24% at 4, 6, and 8 pm, respectively, for TRAV and 28%, 25%, and 24% for BIM.

### Safety

One serious AE (a fall from a gurney in the emergency room) was reported in a patient in the TRAV/BIM group and was not considered to be related to study drug. Ten treatment-emergent AEs were reported with TRAV or BIM (TRAV, n = 5; BIM, n = 5) in 6 patients (TRAV, n = 2/84 [2%]; BIM, n = 4/84 [5%]). Eye disorders were the most common class of treatment-emergent AEs overall (TRAV, n = 1 [1%]; BIM, n = 3 [4%]; Table 2). There were no substantial changes in BCVA from baseline to week 6 for either treatment. No clinically significant alterations in slit-lamp parameters were observed in either treatment group (Table 3). The incidence of abnormal findings of the lens (27% each for TRAV and BIM) and conjunctiva (TRAV, 5%; BIM, 6%) was similar between groups.

**Table 2:** **Treatment-emergent adverse events (safety population)**

	TRAV	BIM
	(n = 84)	(n = 84)
Total TEAEs, n*	5	5
Patients with ≥1 TEAE, n (%)	2 (2)	4 (5)
TEAEs, n (%)		
Chalazion	1 (1)	0
Allergic conjunctivitis	0	1 (1)
Eye irritation	0	1 (1)
Vitreous floater	0	1 (1)
Bronchitis	1 (1)	0
Upper respiratory tract infection	0	1 (1)
Urinary tract infection	1 (1)	0
Fall	1 (1)	0
Panic attack	1 (1)	0
Face swelling	0	1 (1)

BIM = bimatoprost 0.01%; TEAE = treatment-emergent adverse event; TRAV = benzalkonium chloride–free travoprost 0.004%.

*Reflects all adverse events for all patients.

**Table 3:** Abnormal ocular signs* (safety population)

Ocular sign, n (%)		Week 12	
	Baseline	TRAV (n = 84)	BIM (n = 84)
Lens	46 (27)	44 (27)	44 (27)
Cornea	16 (10)	13 (8)	16 (10)
Conjunctiva	8 (5)	8 (5)	10 (6)
Lids/lashes	0	1 (1)	1 (1)
Anterior chamber	0	0	0
Iris	0	0	0

BIM = bimatoprost 0.01%; TRAV = benzalkonium chloride–free travoprost 0.004%.

*Ocular signs evaluated via slit-lamp examination.

At baseline, 93% of patients (n = 77/83) were scored with “none/trace” hyperemia in the study eye, and 7% (n = 6/83) were scored with mild conjunctival hyperemia. After 6 weeks of treatment with TRAV, 69% of patients (n = 29/42) were scored with “none/trace” hyperemia in the study eye. Mild hyperemia was observed in 31% of patients receiving TRAV (n = 13/42), 1 of whom had mild hyperemia at baseline. After 6 weeks of treatment with BIM, 59% of patients (n = 24/41) were scored with “none/trace” hyperemia in the study eye. Mild hyperemia was observed in 39% of patients receiving BIM (n = 16/41), with 4 patients having displayed mild hyperemia at baseline. Moderate hyperemia was reported by 2% of patients (n = 1/41) receiving BIM and 0% of patients receiving TRAV. All incidences of hyperemia were considered to be related to treatment. No incidences of severe hyperemia were reported.

## Discussion

This prospective crossover study was the first to compare the late-day IOP-lowering efficacy and tolerability of BAK-free TRAV and BAK 0.02%–preserved BIM in patients with open-angle glaucoma or ocular hypertension. Mean IOP after 6 weeks of treatment with TRAV was similar to that observed with BIM, indicating that TRAV is noninferior to BIM for IOP lowering. Mean IOP after 6 weeks of therapy ranged from 17 to 18 mmHg (a reduction of approximately 6.0 mmHg from baseline) in both treatment groups. TRAV and BIM were both well tolerated, and no substantial changes were observed in BCVA or ocular signs with either TRAV or BIM. After 6 weeks of treatment, the incidence of mild hyperemia was greater for patients receiving BIM (39%) compared with those receiving TRAV (31%); in addition, the incidence of moderate hyperemia was greater with BIM (2%) than with TRAV (0%).

To date, few studies have compared the IOP-lowering efficacy and safety of TRAV with other prostaglandin analogs. In a large, prospective open-label trial in patients who required transition to a different prostaglandin therapy because of tolerability concerns, mean IOP after 3 months of treatment with TRAV (17 mmHg) was the same as that observed during patients’ previous treatment with bimatoprost 0.03% [25]. Studies comparing TRAV with the prostaglandin analog latanoprost have produced somewhat conflicting results [24–26]. In a 3-month, randomized treatment-switching trial (n = 22) [24] and a smaller (n = 20) 3-month, open-label study [26], no substantial between-group differences in IOP-lowering effect were observed when patients were switched from latanoprost 0.005% to TRAV. However, an open-label switching trial in patients who required alternative IOP-lowering therapy because of tolerability issues demonstrated a significant reduction in IOP after 3 months when patients were switched from latanoprost 0.005% to TRAV (n = 476) [25].

The IOP-lowering efficacy of BAK-free TRAV and BAK 0.02%–preserved BIM was similar in this study, and the efficacy of these formulations is similar to that of their more commonly studied formulations, BAK 0.015%–preserved travoprost 0.004% and BAK 0.005%–preserved bimatoprost 0.03%. In 2 meta-analyses, the IOP-lowering effect was comparable among the 3 most common prostaglandins (i.e., travoprost 0.004%, latanoprost 0.005%, and bimatoprost 0.03%) [20, 28]. Because IOP-lowering efficacy is similar among prostaglandin analogs, treatment may be greatly affected by tolerability. Ocular hyperemia is the most common ocular AE observed with prostaglandin analogs [20]. The reduced incidence of mild and moderate hyperemia observed with TRAV versus BIM in the current study is consistent with results from a prospective treatment-switching study that compared TRAV with bimatoprost 0.03% [25]. Furthermore, BAK-preserved eye drops have been associated with increased ocular discomfort and ocular surface irritation compared with BAK-free medications in patients with ocular hypertension or open-angle glaucoma [29]. Given the similar IOP-lowering efficacy and reduced severity of hyperemia with TRAV compared with BIM, patients may benefit from the use of a BAK-free formulation of TRAV.

This study had several limitations. Assessments were performed at 2 study centers by the same investigator, resulting in a relatively limited patient population. Also, IOP was not measured throughout the entire circadian period. Although this study sought to augment current literature, which predominantly assessed IOP lowering during clinic hours (e.g., 8 am to 4 pm), by providing evidence of the IOP-lowering efficacy of TRAV after office hours, measurement of IOP during a complete 24-hour period may have allowed greater comparison. Furthermore, treatment compliance was not assessed in this study.

## Conclusion

This study assessed the efficacy and tolerability of TRAV versus BIM in patients with open-angle glaucoma or ocular hypertension. The IOP-lowering efficacy of TRAV was noninferior to that of BIM, with both treatments reducing IOP by approximately 25% (6 mmHg) at late-day time points. Both treatments were well tolerated, although a greater incidence of mild and moderate hyperemia was observed with BIM.

## Abbreviations

AE: Adverse event
ANOVA: Analysis of variance
BAK: Benzalkonium chloride
BCVA: Best corrected visual acuity
BIM: Bimatoprost 0.01%
IOP: Intraocular pressure
ITT: Intent-to-treat
PP: Per protocol
TRAV: BAK-free travoprost 0.004%.

## References

[1] American Academy of Ophthalmology Glaucoma Panel (2010). Preferred Practice Pattern® Guidelines. Primary Open-Angle Glaucoma.

[2] Kass MA, Heuer DK, Higginbotham EJ, Johnson CA, Keltner JL, Miller JP, Parrish RK, Wilson MR, Gordon MO (2002). The Ocular Hypertension Treatment Study: a randomized trial determines that topical ocular hypotensive medication delays or prevents the onset of primary open-angle glaucoma. Arch Ophthalmol.

[3] The Advanced Glaucoma Intervention Study (AGIS) Investigators (2000). The Advanced Glaucoma Intervention Study (AGIS): 7. The relationship between control of intraocular pressure and visual field deterioration. Am J Ophthalmol.

[4] Collaborative Normal-Tension Glaucoma Study Group (1998). Comparison of glaucomatous progression between untreated patients with normal-tension glaucoma and patients with therapeutically reduced intraocular pressures. Am J Ophthalmol.

[5] Hughes E, Spry P, Diamond J (2003). 24-hour monitoring of intraocular pressure in glaucoma management: a retrospective review. J Glaucoma.

[6] McCarty CA, Mukesh BN, Kitchner TE, Hubbard WC, Wilke RA, Burmester JK, Patchett RB (2008). Intraocular pressure response to medication in a clinical setting: the Marshfield Clinic Personalized Medicine Research Project. J Glaucoma.

[7] Khaw PT, Shah P, Elkington AR (2004). Glaucoma–2: treatment. BMJ.

[8] Camras CB (1995). Mechanism of the prostaglandin-induced reduction of intraocular pressure in humans. Adv Prostaglandin Thromboxane Leukot Res.

[9] Toris CB, Zhan G, Fan S, Dickerson JE, Landry TA, Bergamini MV, Camras CB (2007). Effects of travoprost on aqueous humor dynamics in patients with elevated intraocular pressure. J Glaucoma.

[10] van der Valk R, Webers CA, Schouten JS, Zeegers MP, Hendrikse F, Prins MH (2005). Intraocular pressure-lowering effects of all commonly used glaucoma drugs: a meta-analysis of randomized clinical trials. Ophthalmology.

[11] Aptel F, Denis P (2011). Balancing efficacy and tolerability of prostaglandin analogues and prostaglandin-timolol fixed combinations in primary open-angle glaucoma. Curr Med Res Opin.

[12] Alm A, Grierson I, Shields MB (2008). Side effects associated with prostaglandin analog therapy. Surv Ophthalmol.

[13] Inoue K, Shiokawa M, Higa R, Sugahara M, Soga T, Wakakura M, Tomita G (2012). Adverse periocular reactions to five types of prostaglandin analogs. Eye (Lond).

[14] Woodward DF, Phelps RL, Krauss AH, Weber A, Short B, Chen J, Liang Y, Wheeler LA (2004). Bimatoprost: a novel antiglaucoma agent. Cardiovasc Drug Rev.

[15] Higginbotham EJ, Schuman JS, Goldberg I, Gross RL, VanDenburgh AM, Chen K, Whitcup SM, Bimatoprost Study Group (2002). One-year, randomized study comparing bimatoprost and timolol in glaucoma and ocular hypertension. Arch Ophthalmol.

[16] Cohen JS, Gross RL, Cheetham JK, VanDenburgh AM, Bernstein P, Whitcup SM (2004). Two-year double-masked comparison of bimatoprost with timolol in patients with glaucoma or ocular hypertension. Surv Ophthalmol.

[17] Williams RD, Cohen JS, Gross RL, Liu CC, Safyan E, Batoosingh AL, Bimatoprost Study G (2008). Long-term efficacy and safety of bimatoprost for intraocular pressure lowering in glaucoma and ocular hypertension: year 4. Br J Ophthalmol.

[18] Katz LJ, Cohen JS, Batoosingh AL, Felix C, Shu V, Schiffman RM (2010). Twelve-month, randomized, controlled trial of bimatoprost 0.01%, 0.0125%, and 0.03% in patients with glaucoma or ocular hypertension. Am J Ophthalmol.

[19] Campbell JH, Schwartz G, Labounty B, Kowalski J, Patel VD (2013). Comparison of adherence and persistence with bimatoprost 0.01% versus bimatoprost 0.03% topical ophthalmic solutions. Curr Med Res Opin.

[20] Li N, Chen XM, Zhou Y, Wei ML, Yao X (2006). Travoprost compared with other prostaglandin analogues or timolol in patients with open-angle glaucoma or ocular hypertension: meta-analysis of randomized controlled trials. Clin Experiment Ophthalmol.

[21] Hollo G, Vargha P, Kothy P (2005). Influence of switching to travoprost on intraocular pressure of uncontrolled chronic open-angle glaucoma patients compliant to previously-used topical medication. Curr Med Res Opin.

[22] Kaback M, Geanon J, Katz G, Ripkin D, Przydryga J, START Study Group (2004). Ocular hypotensive efficacy of travoprost in patients unsuccessfully treated with latanoprost. Curr Med Res Opin.

[23] Lewis RA, Katz GJ, Weiss MJ, Landry TA, Dickerson JE, James JE, Hua SY, Sullivan EK, Montgomery DB, Wells DT, Bergamini MV, Travoprost BAC-free Study Group (2007). Travoprost 0.004% with and without benzalkonium chloride: a comparison of safety and efficacy. J Glaucoma.

[24] Aihara M, Oshima H, Araie M, EXTraKT Study Group (2013). Effects of SofZia-preserved travoprost and benzalkonium chloride-preserved latanoprost on the ocular surface – a multicentre randomized single-masked study. Acta Ophthalmol.

[25] Henry JC, Peace JH, Stewart JA, Stewart WC (2008). Efficacy, safety, and improved tolerability of travoprost BAK-free ophthalmic solution compared with prior prostaglandin therapy. Clin Ophthalmol.

[26] Miyashiro MJ, Lo SC, Stewart JA, Stewart WC (2010). Efficacy, safety, and tolerability of travoprost 0.004% BAK-free versus prior treatment with latanoprost 0.005% in Japanese patients. Clin Ophthalmol.

[27] Dubiner HB, Noecker R (2012). Sustained intraocular pressure reduction throughout the day with travoprost ophthalmic solution 0.004%. Clin Ophthalmol.

[28] Eyawo O, Nachega J, Lefebvre P, Meyer D, Rachlis B, Lee CW, Kelly S, Mills E (2009). Efficacy and safety of prostaglandin analogues in patients with predominantly primary open-angle glaucoma or ocular hypertension: a meta-analysis. Clin Ophthalmol.

[29] Pisella PJ, Pouliquen P, Baudouin C (2002). Prevalence of ocular symptoms and signs with preserved and preservative free glaucoma medication. Br J Ophthalmol.

[CR30] The pre-publication history for this paper can be accessed here: http://www.biomedcentral.com/1471-2415/14/151/prepub

